# Supplementary material for: DMRforPairs: identifying Differentially Methylated Regions between unique samples using array based methylation profiles
Source: BMC Bioinformatics. 2014 May 15;15:141. doi: 10.1186/1471-2105-15-141 (PMC4046028; doi:10.1186/1471-2105-15-141)
Supplement: Additional file 2 — DMRforPairs output for the comparison of A431-MCF7 and NA17018-NA17105. Please start from the HTML files in each folder. Available via the BMC Bioinformatics website. [file 1471-2105-15-141-S2.zip › 1394847754114233_MOESM2_ESM/A431_MCF7/figures/28339.pdf]

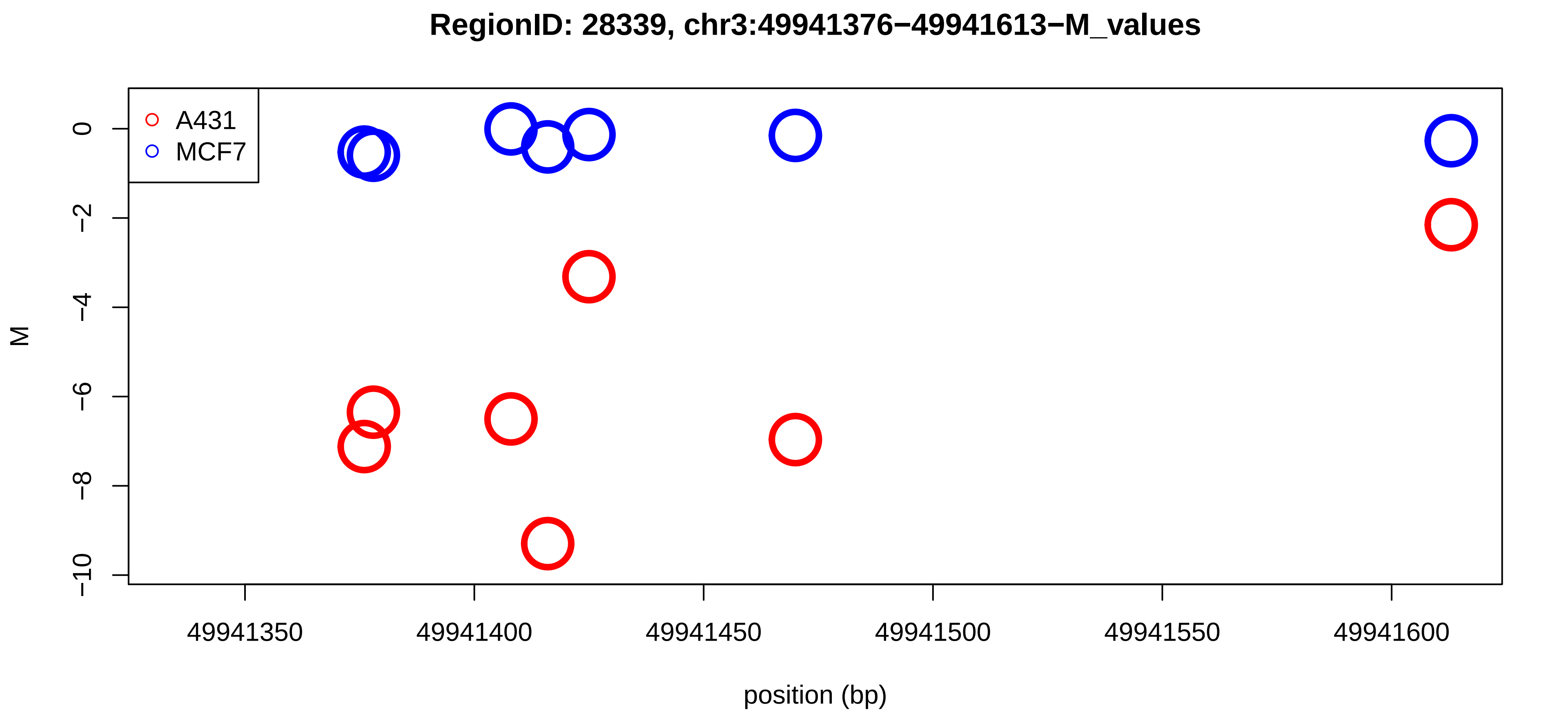
RegionID: 28339, chr3:49941376−49941613−M_values
A431
MCF7
M
0
−2
−4
−6
−8
−10
49941350
49941400
49941450
49941500
49941550
49941600
position (bp)

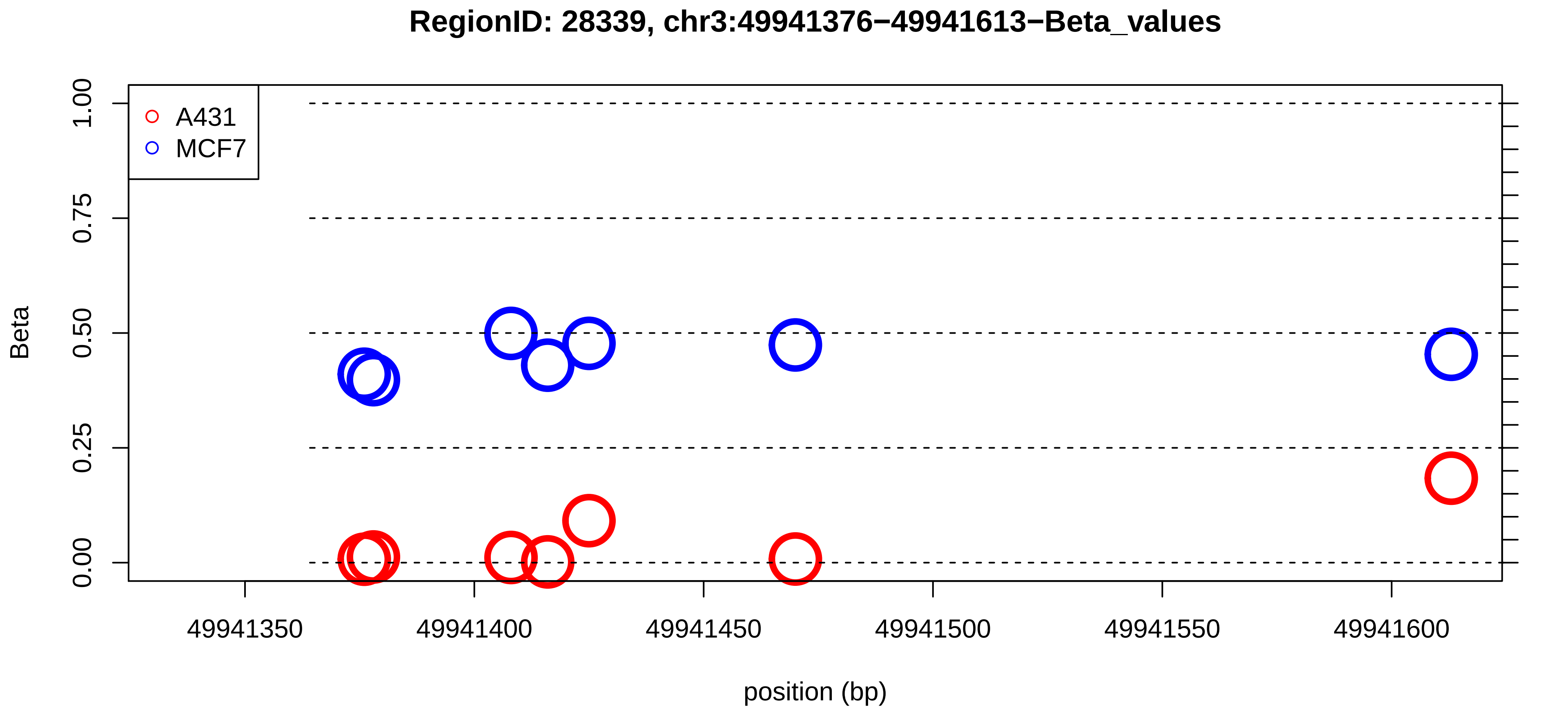
RegionID: 28339, chr3:49941376−49941613−Beta_values
A431
MCF7
Beta
0.00
0.25
0.50
0.75
1.00
49941350
49941400
49941450
49941500
49941550
49941600
position (bp)

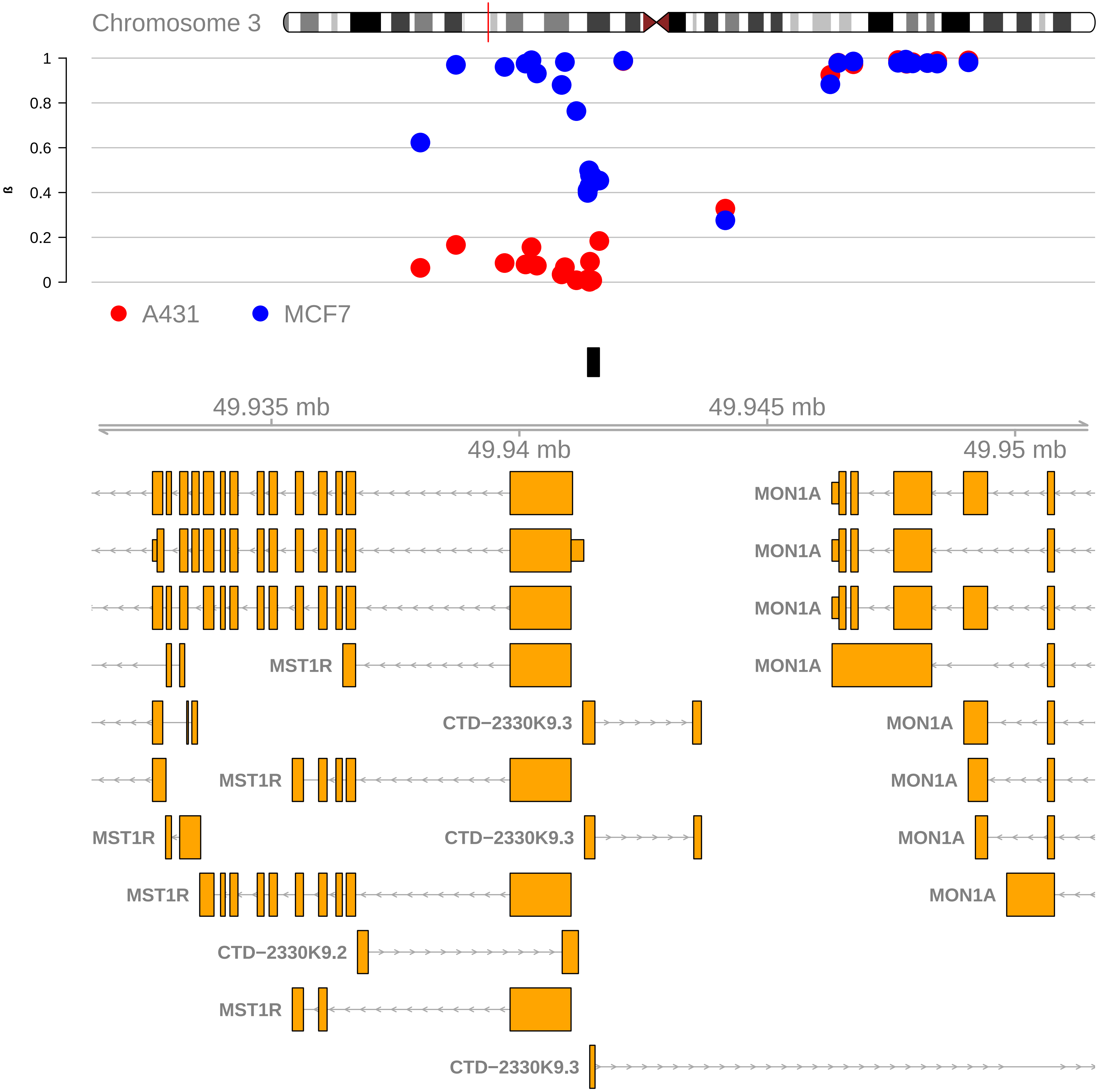

Chromosome 3
1
0.8
0.6
0.4
0.2
0
ß
A431
MCF7
49.935 mb
49.945 mb
49.94 mb
49.95 mb
MON1A
MON1A
MON1A
MST1R
MON1A
CTD−2330K9.3
MON1A
MST1R
MON1A
MST1R
CTD−2330K9.3
MON1A
MST1R
MON1A
CTD−2330K9.2
MST1R
CTD−2330K9.3
